# Assessment of knowledge in oncology about care for transgender people: a scoping review

**DOI:** 10.1590/0034-7167-2023-0532

**Published:** 2024-11-29

**Authors:** Fernanda Fachetti Xavier de Almeida, Cremilson de Paula Silva, Ricardo Souza Evangelista Sant’Ana, Ruan Nilton Rodrigues Melo

**Affiliations:** I Fundação Antônio Prudente A.C.Camargo Cancer Center. São Paulo, São Paulo, Brazil.; II Universidade Federal de Alfenas. Alfenas, Minas Gerais, Brazil.; III Universidade de São Paulo. Ribeirão Preto, São Paulo, Brazil.; IV Universidade de São Paulo. São Paulo, Brazil.

**Keywords:** Transgender People, Health Services for Transgender People, Neoplasms, Oncology, Professional Training, Personas Transexuales, Servicios de Salud para Personas Transexuales, Neoplasias, Oncología, Formación Profesional

## Abstract

**Objective:**

to identify evidence available in the literature on instruments and methodologies used to assess healthcare professionals’ knowledge about cancer care for the transgender population.

**Methods:**

a scoping review was conducted in seven databases, including studies that answered the question: what is the healthcare professionals’ level of knowledge about cancer care for the transgender population?

**Results:**

forty-one articles were selected that dealt specifically with healthcare professionals’knowledge in relation to care for the LGBTQIAPN+ population, especially the transgender population. Eighteen studies assessed patients’ perceptions of professionals’knowledge, whereas other studies used their own assessment tools, considering the global context of LGBTQIAPN+ health.

**Conclusions:**

there is no tested and validated instrument that assesses the knowledge about the transgender population’s oncological health, highlighting the need to construct and validate an instrument focused on this population’s needs.

## RESUMEN

**Objetivo:**

identificar la evidencia disponible en la literatura sobre los instrumentos y las metodologías utilizados para evaluar el conocimiento de los profesionales de la salud sobre la atención oncológica a la población transexual.

**Métodos:**

revisión exploratoria realizada en siete bases de datos, incluyendo estudios que respondieran a la pregunta: ¿Cuál es el nivel de conocimiento de los profesionales de la salud sobre la atención oncológica a la población transexual?

**Resultados:**

se seleccionaron 41 artículos que trataban específicamente sobre los conocimientos de los profesionales sanitarios en relación con la atención a la población LGBTQIAPN+, especialmente a la población transexual. Dieciocho estudios evaluaron las percepciones de los pacientes sobre los conocimientos de los profesionales, mientras que otros estudios utilizaron sus propias herramientas de evaluación, teniendo en cuenta el contexto global de la salud LGBTQIAPN+.

**Conclusión:**

no existe un instrumento probado y validado que evalúe el conocimiento de la población transexual sobre salud oncológica, destacando la necesidad de construir y validar un instrumento centrado en las necesidades de esta población.

## INTRODUCTION

The term LGBTQIAPN+ and its various variations, which seek to encompass the diversity of existing identities, encompass a heterogeneous group with individuals of different ages, experiences, ethnic, cultural and socioeconomic backgrounds. These identities experience daily discrimination and symbolic violence due to social stigmas. These difficulties result in less demand for routine care and an increased incidence of chronic diseases, including cancer, compared to the general population^(1,2)^.

When approaching the LGBTQIAPN+ community, it is essential to consider the concept of intersectionality, which encompasses the overlap of social identities and systems of oppression. It is essential to direct attention to the most vulnerable groups within this minority. Among these groups, transgender individuals emerge as particularly marginalized^(3)^.

The term “transgender” refers to individuals whose gender identity or expression differs from that assigned at birth. Gender identity is a personal experience, encompassing the individual sense of body and various expressions of gender, not always visible to others. The lack of data collection on sexual orientation and gender identity in health instruments makes it difficult to assess the real impact of cancer on the transgender and LGBTQIAPN+ population as a whole^(4)^. However, there is evidence that transgender people disproportionately face modifiable risk factors for cancer, such as smoking, obesity, exposure to viruses associated with sexual behaviors, such as HPV and HIV, in addition to the lack of specialized services in transgender health and inadequate cancer screening^(2)^.

The lack of adequate cancer screening, combined with the increase in risk factors, results in a higher incidence of cases diagnosed in advanced stages, harming the health prognosis of this marginalized population^(4)^.The literature highlights three essential issues related to current knowledge about cancer in LGBTQIAPN+ people: the lack of data collection on sexual orientation and gender identity; the absence of a culturally competent workforce and healthcare system; and the scarcity of information educational programs aimed at this population^(4)^. Cultural competence encompasses knowledge, inclusive attitudes and communication skills, being fundamental in this context^(4)^.

The Brazilian National LGBT Comprehensive Health Policy (*Política Nacional de Saúde Integral LGBT*), established in 2011 by Ordinance 2.836, seeks to combat institutionalized LGBTphobia, ensuring compliance with the Brazilian Health System (SUS – *Sistema Único de Saúde*) universality, equity and comprehensiveness principles. However, its specific guidelines for preventing cancer in trans and transvestites lack clear guidance on how this screening should be carried out^(5)^.

Challenges in prevention for the trans population also include the heteronormativity prevalent in cancer screening campaigns, evidenced by the symbolic use of a pink ribbon for breast cancer and a blue ribbon for prostate cancer. Additionally, cancer treatment spaces reflect “gender” standards, inadvertently discriminating against transgender individuals^(3)^. Using the social name, guaranteed by the Brazilian National LGBT Comprehensive Health Policy, often encounters resistance in practice, generating embarrassment and emotional stress^(5)^.

Research from the National Center for Transgender Equality reveals that almost a third of respondents faced verbal, moral and/ or physical harassment when showing identification documents with a name or sex that disagree with their gender presentation, highlighting the urgent need for more inclusive healthcare environments and respectful^(6)^.

The main challenge for the LGBTQIAPN+ population in accessing healthcare is the fear of discrimination by professionals, often based on previous negative experiences. The 2015 US Transgender Survey revealed that 33% of transgender respondents faced verbal harassment, refused treatment, or had to educate caregivers about transgender issues, whereas 23% avoided medical appointments due to fear of mistreatment^(6)^. The literature highlights the general lack of cultural competence of healthcare professionals in dealing with sexual and, mainly, gender minorities, with the minimum workload and restricted focus on topics such as sexual health, sexually transmitted infections and HIV in health courses directly contributing for this deficiency^(2,4)^.

## OBJECTIVE

To identify and map scientific evidence available in the literature on instruments and methodologies used to assess healthcare professionals’knowledge about oncological care for the transgender population.

## METHODS

### Ethical aspects

As this was a review study, there was no need for assessment by a Research Ethics Committee, in accordance with the Sole Paragraph of Article 1 of Brazilian National Health Council Resolution 510/16, item VI. Furthermore, it is noteworthy that the studies selected for the final sample were duly referenced.

### Study design

The study was conducted in accordance with the methodological structure adopted by JBI, consisting of nine steps for scoping review^(7,8)^, as well as the Preferred Reporting Items for Systematic Reviews and Meta-Analyses extension for Scoping Reviews (PRISMA-ScR)^(9,10)^. It is noteworthy that all authors underwent training at JBI on scoping review before the start of the study, which took place from May 6, 2023 to July 22, 2023.

### Protocol and registration

The study protocol was registered through the Open Science Framework (OSF) platform with DOI registration: 10.17605/OSF. IO/EFQAN.

### Research question

To develop the research question, the PCC strategy (Population, Concept and Context) was used. It was defined: P – Population: healthcare professionals; C – Concept: level of knowledge and C – Context: oncological care for transgender people. Therefore, the guiding question of the research s: what is the healthcare professionals’ knowledge level regarding oncological care for the transgender population?

### Search strategy and data sources

The search strategy was developed with the help of a librarian (JDLA) from the main investigator’s institution, using controlled and uncontrolled descriptors from Medical Subject Headings (MeSH), Cumulative Index to Nursing and Allied Health Literature (CINAHL) and Health Sciences Descriptors (DeCS). The most sensitive and reliable descriptors were selected. To develop the final search strategy, terms related to the acronym PCC were used, combined with Boolean operators AND and OR. After testing the search strategies in the seven databases, such as CINAHL, PubMed (National Library of Medicine), EMBASE (Elsevier Science), LILACS (Latin American and Caribbean Literature in Health Sciences), Scopus (The Search Portal for Life Sciences), WoS (Web of Science) and COCHRANE (Cochrane Central Register of Controlled Trials), the same search strategy was standardized for all bases: (“Cancer Care” OR “Cancer Care Facilities” OR “Cancer” OR “Neoplasms” OR “Oncology”) AND (“Health Services for Transgender Persons” OR “Transgender Persons” OR “Transgender”) AND (“Professional”).

### Selection criteria

The search was carried out on May 6, 2023 by two independent authors (FFXA, CPS). No language filters or publication period were selected. All types of studies were included, as long as: I) they involved an approach to the transgender population; II) they carried out an assessment of healthcare professionals’knowledge/ preparation level in the oncological context.

### Selection of sources of evidence

Access to data sources was through the Coordination for the Improvement of Higher Education Personnel (CAPES – *Coordenação de Aperfeiçoamento de Pessoal de Nível Superior*) Portal Journal.

### Data mapping and extraction

**Figure 1:**
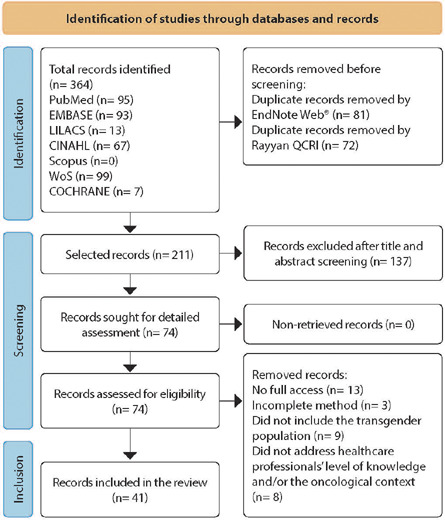
Study search flowchart based on the Preferred Reporting Items for Systematic reviews and Meta-Analyses extension for Scoping Reviews, 2023

The titles and abstracts retrieved in the searches were grouped in the EndNote Web® reference management database to identify and exclude duplicates. To select and assess the sample studies, the Rayyan QCRI platform was used. Two reviewers (FFXA, CPS) independently read and analyzed the titles and abstracts, ensuring that the process was carried out blindly using the blind on feature available in the software, in order to identify studies relevant to the guiding question of this research. Disagreements regarding the study assessment process among reviewers were resolved by a third reviewer (RNRM). After the search, articles that were not available in full were identified and, to retrieve them, up to three attempts were made to contact the authors. Figure 1 shows the number of articles found and included presented by the PRISMA-ScR flowchart.

### Data analysis and presentation

After analyzing the studies that made up the final sample (FFXA, CPS, RNRM and RSES), it was possible to categorize them into five axes, namely: Instruments used to assess healthcare professionals’ knowledge regarding care for LGBTQIAPN+ patients with cancer (this category included studies that used an instrument that assessed healthcare professionals’ oncological knowledge about the LGBTQIAPN+ population); Perception of LGBTQIAPN+ patients with cancer regarding healthcare professionals’knowledge (this category included studies that assessed what perception LGBTQIAPN+ patients with cancer had regarding professionals’knowledge); Incipient academic training: difficulties in providing comprehensive care for the LGBTQIAPN+ population (this category included studies that, despite assessing knowledge, addressed the weaknesses found in the educational system in health areas); Nursing Process implementation: weaknesses in care (this included studies that addressed the challenges of nursing in assisting people belonging to sexual and gender minorities); Comprehensive care: strategies to offer a comprehensive approach to the needs and demands of LGBTQIAPN+ patients with cancer (the studies included in this category addressed information on possible strategies to improve care for LGBTQIAPN+ patients).

## RESULTS

The search strategies allowed us to find 364 articles, of which 81 replicates were eliminated using EndNote Web^®^ reference manager software. Subsequently, the remaining studies from this initial analysis were transferred to the database created in Rayyan QCRI application, where it was possible to eliminate another 72 duplicate studies. After assessment by a third reviewer, 74 articles were selected to be assessed in full and analyzed according to pre-established eligibility criteria. Of these, 41 articles specifically dealt with healthcare professionals’knowledge in relation to care for the LGBTQIAPN+ population, especially the trans population.

Psychosocial health is influenced by a complex intersection of demographic, clinical and social factors in which individuals are included^(11)^. Compared to cisgender heterosexual groups with cancer, sexual and gender minorities with cancer exhibit significant sociodemographic, psychosocial, and clinical differences, reflecting unique experiences shaped by systemic factors that marginalize Queer identities and favor cisgender and heterosexual identities^(11)^. This means that these minorities experience the health-disease process in different ways, including within the LGBTQIAPN+ subgroups, requiring different care^(11)^. The studies raised in this review address professionals’ knowledge about these differences.

As for the place of publication of the research, it was identified that North America and Europe were largely responsible for the content produced. The United States led in number of studies, with 31 studies (78%), followed by the United Kingdom, with six (15%), Australia, with two (5%), Switzerland and Canada, with one study each, respectively.

Regarding the studies’methodological approach, a variety of research designs was observed. A total of 15 studies were conducted with a qualitative approach (36.59%), indicating a strong presence of exploratory and descriptive research. Eleven review studies were identified: five were classified as systematic reviews (12.20%); four were classified as narrative reviews (9.76%); and two were classified as scoping reviews (4.88%). Ten studies used a cross-sectional design (24.39%) and five studies were classified as quasi-experimental (12.20%).

When it comes to the year of publication of the 41 studies that made up the sample of this review, there was a predominance of studies in 2022, with nine articles (21.95%), followed by 2018 and 2021, with seven studies each (17.07%). In the studies analyzed, the most used methodological design was qualitative, in 16 studies (39.02%), followed by cross-sectional quantitative studies, in ten studies (24.39%), as well as review studies, respectively.

As for the target population, 20 studies focused only on healthcare professionals, of which nine only addressed specialists and/or professionals who worked in oncology services, and only one addressed undergraduate health students. Two studies were developed in cancer treatment centers, whereas the others did not make distinctions regarding the level of healthcare in which professionals worked. Thirteen studies involving the LGBTQIAPN+ population were included: six of them only addressed patients with cancer, one of which focused exclusively on cancer survivors; two studies also included caregivers of these patients; a study involved advocates and researchers on issues related to the LGBTQIAPN+ population. Only six studies focused exclusively on the transgender population, four of which were in the oncological context, which highlights the lack of research that addresses the specificities of this population. Two studies addressed both professionals’and patients’perspectives. Although some studies mentioned challenges related to financial difficulties and health insurance coverage, none specified whether the discussions dealt with public or private healthcare services.

**Chart 1:** Distribution of studies according to article code, main author, year of publication, category in which they were included and main recommendations found, 2023

Code/author/year	Study category	Main recommendations
A1/ Banerjee *et al*., 2018	Category 1	A needs assessment survey is recommended to identify professionals’ difficulties in communicating with LGBT patients and their needs, aiming to develop sensitivity training.
A2/ Habib *et al*., 2023	Category 1	New research to improve scales to compare providers’ and patients’ experiences with LGBTQI-affirming healthcare. The scales in this study need to be assessed for possible improvements, making them less tiring and allowing the reporting of variations in care experiences by professionals.
A3/ Shi *et al*., 2023	Category 1	It is necessary for institutions to assess the cultural competency of perioperative clinical staff in relation to transgender health, especially within certain demographic groups.
A4/ Shires *et al*., 2019	Category 1	Include training in inclusive healthcare for transgender people as a routine part of the training of all healthcare professionals, also addressing personal attitudes.
A5/Sonnenblick *et al*., 2022	Category 1	In breast imaging practice, there is a substantial need to record transgender and other gender nonconforming information.
A6/ Sutter *et al*., 2020	Category 1	It is important to continually emphasize the importance of sexual orientation and gender identity data collection to support educational efforts and thereby improve the quality of care provided to LGBTQ+ individuals across the cancer continuum.
A7/ Berner *et al*., 2020	Category 1	Awareness of LGBTQ+ care needs in oncology should be integrated into graduate medical and clinical oncology curricula. It is recommended to create an online repository of existing educational material, supplemented by UK-specific content. It is crucial to conduct high-quality research to collect data on sexual orientation and gender identity to understand cancer differences, risks, and outcomes in these populations.
A8/ Shetty *et al*., 2016	Category 1	Large national surveys are needed to determine whether the knowledge, attitudes, and practice behaviors identified in this study are representative of healthcare providers in the United States. A nationally representative sample is crucial to developing training and curricula that address identified gaps.
A9/ Zayhowski *et al*., 2019	Category 1	The findings of this study point to opportunities for the field of genetic counseling to improve services for transgender patients by reporting distinct situations that may arise in the clinic with these patients and providing training recommendations for genetic counselors.
A10/ Ussher *et al*., 2022	Category 1	Systemic changes are needed to overcome barriers to providing culturally competent oncology care for LGBTQI patients. Therefore, it is recommended to include content both for the LGBTQI community in general and specific to each subgroup within that community and in education and professional training curricula.
A11/ Unger *et al*., 2015	Category 1	Efforts should be made to educate trainees about important aspects of transgender care, and comprehensive guidelines for providers should be published.
A12/ Gatos *et al*., 2018	Category 2	Invest in research that analyzes the experiences and needs of transgender men so that specific recommendations and guidelines can be reached for this population.
A13/ Cathcart *et al*., 2020	Category 2	Assess a patient’s comfort level with discussions about sex and sexuality initially and periodically to ensure a satisfactory quality of sexual life for patients.
A14 / Kamen *et al*., 2019	Category 2	Studies focused on the individual experiences of transgender, non-binary, and black patients with a current cancer diagnosis are needed as well as mixed methods studies to examine disparities in oncology care for LGBTQI patients.
A15/ Cloyes; Candrian, 2021	Category 2	Palliative care services must collect sensitive data about patients’ sexual orientation and gender identity. Additionally, it is critical to provide adequate training and education about the individual needs of LGBTQ+ patients and create safe and representative environments for these vulnerable populations.
A16/ Kamen, 2018	Category 2	Ensure cultural competence among healthcare professionals regarding the LGBT population; have reference services for reception; analyze and review social support programs; and conduct education through community organizations.
A17/ Kerr *et al*., 2021	Category 2	It is necessary to create education programs in partnerships with organizations that support transgender health, create inclusive policies and improve data collection for cancer epidemiology in transgender people.
A18/ Power *et al*., 2022	Category 2	It is recommended to invest in welcoming and safe environments, focusing on assessing and increasing professionals’ knowledge about LGBTQI health. It also suggests the importance of having professionals who are part of this community as care providers.
A19/PrattChapman *et al*., 2021	Category 2	It is recommended that there be clear explanations and open conversations with patients, in addition to the development of specific screening guidelines for sexual and gender minorities.
A20/ Sledge, 2019	Category 2	The study recommends that institutions create actions and research that contribute to developing patientcentered care in order to allow patients to express their wishes and desires.
A21/ Stenzel *et al*., 2020	Category 2	Provide educational training to clinicians on gender and sexual minority care, recognizing disparities in suffering in vulnerable populations.
A22/ Rolle *et al*., 2022	Category 2	Improvements in the educational training system for healthcare professionals are recommended as well as investigations into screening, surveillance and experiences of transgender men in relation to cancer.
A23/ Webster *et al*., 2021	Category 2	Education in relation to appropriate languages and pronouns and proposals for education in oncology and radiotherapy from the perspective of disparities; creation of support groups; collecting data on sexual orientation and gender identity and investing in research in the area.
A24/ Kerr *et al*., 2021	Category 2	Oncology nurses need to reflect on their own assumptions regarding gender issues and strengthen ties for open communication with their patients.
A25/ Floyd, *et al*., 2020	Category 2	It is necessary to improve undergraduate and graduate curricula with the inclusion of sexual and gender diversity topics for all healthcare professionals, in addition to the adoption of inclusive language and a welcoming environment.
A26/ Lauren *et al*., 2016	Category 2	Review of curricular bases for medicine and health courses and increased interactions with this population, from communication to relationships with people.
A27/ Bristowe *et al*., 2018	Category 2	Use appropriate language, explore care for the intimate region of transgender patients, help create support and inclusion policies, and increase the visibility of LGBT people in institutional materials.
A28/ Squires *et al*., 2022	Category 2	Studies should delve deeper into biopsychosocial issues with interinstitutional collaborations, with a greater focus on the most vulnerable groups, which are transgender people. Create a welcoming environment and promote specific education for patients and professionals.
A29/ Gannon *et al*., 2022	Category 2	The use of appropriate terminologies and language is recommended, in addition to dissemination and education on the topic. Furthermore, institutional issues of reception and belonging must be implemented to improve patient experience.
A30/ Burgart *et al*., 2022	Category 3	Implement a more uniform and reliable distribution method by sending surveys directly to residents or including a limited number of questions in the annual in-service exam for all resident examinees. Measuring this data could improve resident education, especially after implementing specific interventions.
A31/ Hunt *et al*., 2019	Category 3	Better training of health and social care staff is needed. Training materials that incorporate more evidencebased attitude and behavior change techniques should be developed and then assessed to ensure their effectiveness with health and social care staff in a wide range of contexts.
A32/PrattChapman *et al*., 2023	Category 3	Competency training for radiologists to care for the transgender and intersex population covering terminology, physical examination, radiotherapy adaptations, potential side effects for patients with pervasive developmental and intersex disorders, personal experiences in caring for these minorities, clinical recommendations and care coordination.
A33/ Damascos *et al*., 2018	Category 4	Expanded investigation into LGBT issues, including intersectional identities and social determinants that affect cancer prevention, screening, treatment, and survival. Additionally, it is essential to increase access to cancer screenings and inclusive education for all LGBT people, promoting positive health behaviors. Training based on cultural humility and intersectionality is needed to enable professionals to provide compassionate care to LGBT people with cancer.
A34/ Callahan *et al*., 2015	Category 4	Lessons learned to improve care for LGBT patients can be applied to efforts to improve care for all marginalized groups. To make changes in the delivery of healthcare, individuals and institutions need to make a long-term commitment to change.
A35/ Cloyes *et al*., 2018	Category 5	Implementation of the diversity topic in training curricula; creation of support and guidance resources; guarantee of knowledge assessment; and ongoing skills training.
A36/ Chidiac *et al*., 2021	Category 5	Develop educational training and knowledge assessment in partnership with different sectors and teams in the health sector.
A37/PrattChapman, 2022	Category 5	Develop studies that use validated measures to assess cultural competence and professionals’ knowledge about oncology applied to the transgender population.
A38/PrattChapman, Phillips, 2020	Category 5	Develop continuing education strategies aimed at diversifying and preparing professionals to meet LGBT patients’ demands, in addition to the importance of assessing their knowledge.
A39/PrattChapman. 2021	Category 5	Promote opportunities for self-reflection among professionals, in addition to structuring educational strategies that are interactive to improve knowledge.
A40/ Block *et al*., 2022	Category 5	Training implementation strategies, in addition to being validated with professionals, recommend that they be validated with patients of different sexual orientations and gender identities to ensure greater representation in training.

## DISCUSSION

### Instruments used to assess healthcare professionals’knowledge regarding care for LGBTQIAPN+ patients with cancer

Eleven selected studies focused on assessing healthcare professionals’ knowledge and/or perception.

Most researchers have structured their own assessment instruments to achieve specific objectives. Sonnenblick *et al*. (2022)^(12)^ created an instrument to assess practice and needs in the area of breast imaging for transgender individuals, whereas Shi *et al*. (2023)^(13)^ focused on perioperative healthcare professionals’attitude, knowledge and behavior in relation to trans people’s health.

Other studies used personalized questionnaires to examine the importance of healthcare professionals’knowledge about the specific needs of LGBTQIAPN+ patients, highlighting the need for more focused attention on communication, beliefs and behavior from oncology healthcare providers for this population^(14 ^–^ 20)^.

Habib *et al*. (2023)^(21)^ validated a specific instrument to assess affirmative care for LGBTQIAPN+ patients. The study developed two scales, QUIRKS-Provider and QUIRKS-Patient, to assess clinical environment and behaviors. The scale for professionals assessed objective knowledge and self-reported preparedness, whereas the scale for patients assessed care experiences and satisfaction.

The Lesbian, Gay, Bisexual, and Transgender Development of Clinical Skills Scale (LGBT-DOCSS) is the only validated scale to assess self-reported knowledge, skills, attitudes, and preparation by healthcare professionals in caring for LGBT people. This scale is used by Habib *et al*. (2023)^(21)^ and other studies, but it is generic and does not specifically address cancer, nor subpopulations, such as transgender people.

Some studies opted for qualitative approaches, such as semistructured interviews, to obtain a more subjective view. Zayhowski *et al*. (2019)^(22)^ explored oncology genetic counselors’experiences in caring for transgender patients. Several studies have highlighted the need for ongoing training and education to improve healthcare professionals’knowledge and attitudes^(17 ^–^ 19)^. This highlights the importance of including these topics in training curricula and implementing specific and ongoing educational strategies for more inclusive and sensitive care delivery to this population.

Some studies have identified challenging attitudes and behaviors that reflect a lack of sensitivity toward LGBTQIAPN+ patients, such as reluctance to provide routine care, such as cervical smear screening to trans male individuals^(13,16)^.

A study with a qualitative design, carried out through interviews, highlighted interpersonal challenges in the interaction between healthcare professionals and transgender patients, indicating the need not only for technical knowledge, but also for specific interpersonal skills to provide inclusive care^(22)^.

### Perception of LGBTQIAPN+ patients with cancer regarding healthcare professionals’ knowledge

In this category, 18 studies that address healthcare professionals’knowledge from the perspective of patients with cancer were included for analysis. All studies included in this category indicated the need for adequate training for healthcare professionals^(3,23,24,25,26,27,28,29,30,31,32,33,34,35,36,37,38)^. The majority highlighted the weakness in specific knowledge, skills and approach to the LGBTQIAPN+ population, especially the transgender population^(3,23,24,25,26,28,29,30,31,33,34,35,36)^. Studies have shown that healthcare professionals are unprepared to care for patients, offering low-quality care due to the lack of complete and pertinent information, which constitutes a barrier to obtaining comprehensive care^(3,23,28,30,31,33,36,38)^.Furthermore, studies highlighted that professionals do not have knowledge of how to question patients about their gender identity and sexuality^(3,23,26,28,30,32,33,34,35,38)^. Lack of understanding about the concept of transsexuality, the transition process, and misinterpretation of sexuality, gender, assumptions, and health-related issues were identified as barriers to access for the transgender population, resulting in negative experiences when seeking healthcare, making them feel “invisible” and with their needs unmet^(32,34,35)^.

Furthermore, the research analyzed indicates that the lack of awareness, empathy and understanding of the individual needs of LGBTQIAPN+ patients contributes to the emergence of fear, anguish, emotional and psychological stress^(3,23,24,25,26,28,32,33)^. This, in turn, creates barriers to accessing healthcare services, makes it difficult to obtain accurate information about the therapeutic pathway, and can negatively affect interactions with healthcare professionals^(3,23,24,25,26,27,28,29,30,32,34,35,38)^. This interaction is already compromised by the diagnosis of a disease that can interfere with continuity of life and bring emotional, physical, social and spiritual consequences^(36,38)^.

Discrimination and prejudice historically experienced by participants were also highlighted in studies as aggravating factors in the interaction of patients with professionals^(3,24,25,26,27,30,32,33,34,35,36)^. This intrinsic prejudice and stigma in society, together with the lack of knowledge, constitute individual and institutional obstacles^(24)^. To minimize the impacts of this historical fact, studies affirm the need for healthcare providers to make it clear that all forms of discrimination and prejudice will not be tolerated^(25,28,32,35,38)^.

In this context, a study by Kamen *et al*. (2019), with a qualitative approach that addresses the experiences and recommendations of lesbian, gay, bisexual, transgender and queer (LGBTQ) patients with cancer, emphasizes that a comprehensive approach to patients must include negotiating the disclosure of sexual orientation and identity gender based on patient safety^(3,24,25,32,36,38)^. Another review study conducted by Cloyes and Candrian (2021) pointed out that the effects of historical and structural disparities faced by LGBTQIAPN+ patients can be minimized by the knowledge and skills necessary to provide competent care centered on each person’s individuality. This includes the participation of family members and partners as agents in the care process^(24)^.

Support from family members, inclusive support groups and especially the presence of companions during the journey from diagnosis to prognosis were highlighted in most studies as mitigating the negative impacts related to prejudice and lack of knowledge on the part of professionals^(3,24,25,27,31,32,33,34,35,36)^. Furthermore, it was observed that the exclusion of partners in healthcare results in the loss of dignity of patients, negatively interfering in all spheres of their lives^(28,29,32)^.

Following these principles, studies indicated that participants felt uncomfortable in traditional support groups, experiencing feelings such as loneliness and sadness as well as not feeling welcomed to speak freely about issues related to sexual health and morbidity without fear of discrimination^(3,24,25,26,32,33)^. The lack of knowledge regarding the individual and specific needs of LGBTQIAPN+ patients with cancer results in emotional and psychological changes, as highlighted in a study conducted by Webster and Drury-Smith (2021), which highlighted the vulnerability in healthcare services for not offering adequate psychological care for LGBTQIAPN+ patients^(32)^.

Furthermore, through analysis of studies^(3,23,24,25,26,27,28,29,30,31,32,33,34,35,36,37,38)^, the need for permanent and continuous education actions for healthcare professionals is understood to minimize the impacts of historical disparities and improve the provision of services to LGBTQIAPN+ people with cancer, the aim of which is to offer comprehensive, dignified, respectful and qualified care.

### Incipient academic training: difficulties in providing comprehensive care for the LGBTQIAPN+ population

Three studies analyzed healthcare professionals’ knowledge caring for LGBTQIAPN+ patients. The data demonstrate that, despite investments in professional training through undergraduate and/ or graduate programs, patients still feel vulnerable in relation to the care offered by professionals^(39,40)^.

In interviews carried out with 16 professionals who work in oncology services, a weakness in the training process, knowledge and confidence related to the multidimensional aspects of care for transgender people diagnosed with cancer could be identified^(39)^. During students’ training, it was highlighted that information on care for the LGBTQIAPN+ population is limited and does not provide concrete support for implementing qualified care focused on the needs of this population.

Furthermore, a study carried out by Burgart *et al*. (2022) revealed that the majority of resident doctors did not feel prepared to provide care to the transgender population, with many reporting little access to related content during their academic training^(41)^. Unger (2015) also identified that the majority of residents in obstetrics and gynecology lack specific training in healthcare for transgender people, regardless of time in practice^(20)^.

These findings support other studies that demonstrate fragility in the training process, contributing to gaps in establishing bonds with patients and difficulties in offering comprehensive care^(42,43)^. This gap in knowledge regarding care is also attributed to the lack of training and continuing education focused on strategies to approach the transgender population^(44)^.

Therefore, it is essential to include this topic in undergraduate and graduate curricula to promote positive experiences for patients, improving cancer screening actions, quality of life and increasing interaction and satisfaction with health services, as well as with the care offered by professionals working in oncology.

### Nursing Process implementation: weaknesses in care

Two articles addressed the integration of sexual orientation and gender identity in healthcare, highlighting crucial aspects of fragility in care, but with different focuses.

Damaskos *et al*. (2018) highlight the importance of considering intersectionality when caring for LGBTQIAPN+ patients with cancer and discuss the weaknesses in care due to the lack of recognition of these multiple identities^(1)^. They recognize that the cancer experience is shaped by a variety of aspects, such as race, social class and other factors. A sensitive approach to these factors is essential to fully understand the needs and challenges faced by these patients. Neglecting these aspects can result in significant gaps in healthcare.

Callahan *et al*. (2015) support this need, emphasizing the importance of including information about sexual orientation and gender identity in electronic health records to ensure a more comprehensive care provision^(45)^. The study addresses the practical implementation of sexual orientation and gender identity data collection, highlighting the challenges and opportunities of incorporating LGBTQIAPN+ context-sensitive information into electronic health records^(45)^. They emphasize that the lack of adequate infrastructure and institutional culture can represent obstacles to this implementation, requiring adaptations.

These studies emphasize the importance of an inclusive and sensitive approach to the specific needs of LGBTQIAPN+ patients with cancer to improve the quality of healthcare provided.

### Comprehensive care: strategies to offer a comprehensive approach to the needs and demands of LGBTQIAPN+ patients with cancer

In this category, only two articles focused on training professionals to provide palliative and end-of-life care to LGBTQIAPN+ patients with cancer, whereas the others addressed broader training.

Cloyes *et al*. (2018) did not perform any practical interventions, but they offer a comprehensive literature review, highlighting challenges and gaps in palliative care for LGBTQIAPN+ patients, and bring together recommendations for professionals’ daily practice as well as sources of available support resources that aim to promote care more effective and inclusive palliative and end-of-life care^(46)^. Chidiac *et al*. (2021) developed and implemented an educational program for interdisciplinary palliative care teams that consisted of a one and a half hour workshop, aimed at improving LGBTQIAPN+ cultural competence^(47)^. The intervention was delivered in four UK institutions, with a total of 145 individuals (healthcare professionals and social workers), and was assessed through Kotter’s eight-step process for leading change. A non-equivalent pre-posttest quasi-experimental group design was used to measure the impact of the intervention and showed a significant increase in reported levels of knowledge, confidence and comfort with issues, needs and terminology related to the LGBTQIAPN+ population and palliative care after participation in training.

Just as Cloyes *et al*. (2018), Radix and Maingi (2018) also gathered reliable sources of support resources available to promote quality care for sexual and gender minorities^(2)^. The researchers also reviewed the interventions proposed and developed, up to the time of the study, to improve knowledge about LGBT health. The training sessions varied from 45 minutes to ten hours; many used a lecture format, however others included case studies, videos and/or LGBT panels.

Only one article had as its main target the introduction of an LGBTQIAPN+ cultural competency component into an interprofessional curriculum for healthcare students. Pratt-Chapman and Phillips (2020) promoted an eight-hour symposium at George Washington University in the USA seeking to better prepare healthcare professional students and professors to care for sexual and gender minority patients^(48)^. The researchers used the LGBT-DOCSS scale to compare the knowledge, attitudes, and clinical preparation of the students surveyed as well as the perceived value of interprofessional learning before and after the symposium. Moreover, they also compared the post-test results of the participating students with those of an interprofessional group who did not attend the symposium. In contrast to the comparison group, post-test symposium participants obtained higher scores on learning objectives, attitudes and knowledge of LGBT-DOCSS factors, and perceived value of interprofessional learning, as measured by four items from the Readiness for Interprofessional Learning Scale. This result reinforces the benefit of greater curricular integration of sexual and gender minority health content through interprofessional learning to ensure the preparation of all professionals.

Pratt-Chapman also implemented a cultural competence training program for oncology social workers^(49)^, which consisted of a workshop with didactic and interactive content taught to a sample of 26 social workers, in addition to the implementation of cultural training Together for Education About Cancer in Minorities (TEAM)^(50)^. TEAM training is designed to improve health equity in cancer care organizations by guiding teams of interprofessional students through planning and implementing quality improvements to promote equitable, accessible, and patient-centered oncology care. Training components included: a five-hour self-paced online course; three virtual technical assistance sessions to help teams prepare for the in-person portion of training; a two-and-a-half-day in-person workshop focused on developing an organizational action plan to improve a cancerrelated service to be more culturally sensitive and equitable; and three virtual technical assistance sessions to help address action plan implementation challenges. The study compared changes in self-reported cultural competence measured by the Cultural Competence Assessment (CCA), LGBT-DOCSS and Interprofessional Socialization and Valuation Scale (ISVS). The primary objective of the study was to assess changes in self-reported cultural competence, and the secondary objective was to examine changes in interprofessional assessment from baseline to post-intervention.

Block *et al*. (2022) address cultural competence in the specific context of fertility and focus on the population of young adults with cancer^(51)^. The Enriching Communication skills for Healthcare professionals in Oncofertility (ECHO) team created an educational module in response to the needs of oncology allied healthcare professionals to provide inclusive and affirming care to LGBTQidentified young adult patients with cancer.

The articles gathered in this section highlight a notable convergence in recognizing the importance of educational and training strategies to enhance cultural competence and sensitivity to diversity among healthcare professionals caring for LGBTQIAPN+ patients with cancer. Interventions ranged from specific educational programs, such as TEAM’s cultural training^(50)^, to online training modules, such as LOvE ECHO Training^(51)^. The predominant quantitative assessment through questionnaires before and after the interventions allowed the measurement of changes in participants’ attitudes and knowledge.

The studies produced and the trend of scientific production in the area reveal that the main gaps in relation to the transgender population’s oncological health begin in the educational base. Gender identity and sexual orientation are subjects little or not covered in basic training courses and to a lesser extent in higher education courses in health. This means that professionals enter the job market with many prejudices and insecurities regarding the vulnerable populations’ health. When it comes to oncology, the data produced is still focused on the binarism of cisgender gender identities and there is a lack of theories, models and indepth programs that explore individual experiences of the disease in this population, which ends up creating even more challenges for the advancement of transgender health in oncology.

Furthermore, the lack of epidemiological knowledge evidenced in all studies on how cancer behaves in specific vulnerable populations makes it difficult to meet individual, patient-centered healthcare needs.

It is important that scientific research increasingly includes data collection in its studies on gender identity and sexual orientation of people with cancer in order to be able to assess the impacts caused by the repercussions of cancer and its treatments on vulnerable populations such as the transgender population.

### Study limitations

A limitation of this research can be considered the fact that we were unable to access 13 studies, as they were not available in full, even after three attempts to contact the authors.

### Contributions to health, nursing or public policy

Studies as well as instruments aimed at transgender people reside in recognizing that the care offered in the context of oncology nursing may differ significantly from other members of the LGBTQIAPN+ community. Identifying these needs and challenges is essential to promote inclusion and equity in cancer care, which can provide a more welcoming and safer environment for transgender people, contributing to reducing inequalities. This seeks to improve care for the transgender population and, consequently, improve their access to healthcare services. Thus, it is possible to glimpse the contributions of this research regarding the commitment made by nursing in the global context to achieve the Sustainable Development Goals (SDGs) determined by the United Nations (UN), with emphasis on SDG 3, which aims to ensure a healthy lives and promote well-being for all at all ages.

## CONCLUSIONS

Only six studies were aimed exclusively at transgender people, which highlights the need to expand research and knowledge on issues related to cancer among this population. Furthermore, it was not possible to highlight tested and validated instruments to measure transgender population’s oncological health knowledge, which demonstrates a significant gap in the literature consulted, highlighting the urgency of developing and validating specific instruments that can provide an accurate assessment of healthcare professionals’ knowledge about the specific needs of the transgender population in the context of cancer.

It is important to highlight the need for further research aimed at the transgender population in the field of oncology, expanding knowledge and raising awareness about the unique challenges of this community, contributing to improving health systems and equipping professionals with the necessary tools to provide equitable and culturally sensitive care sensitive.

Therefore, the construction and validity of a specific instrument to assess the transgender population’s oncological health knowledge are highly relevant steps to ensure the provision of healthcare in an inclusive and sensitive manner to this culturally vulnerable community. This instrument can facilitate developing targeted and effective interventions and training programs, contributing to reducing disparities in access and quality of cancer care for transgender people.

## References

[1] Rodrigues TS, Sant’Ana RSE, Zerbinati JP, Souza LN, Sousa AR, Maheu C (2023). Approaching sexuality in LGBTQIAP + patients with cancer: scoping review. BMC Public Health.

[2] Radix A, Maingi S (2018). LGBT cultural competence and interventions to help oncology nurses and other health care providers. Semin Oncol Nurs.

[3] Kamen CS, Alpert A, Margolies L, Griggs JJ, Darbes L, Smith-Stoner M (2019). “Treat us with dignity”: a qualitative study of the experiences and recommendations of lesbian, gay, bisexual, transgender, and queer (LGBTQ) patients with cancer. Support Care Cancer.

[4] Margolies L, Brown CG (2018). Current State of Knowledge About Cancer in Lesbians, Gay, Bisexual, and Transgender (LGBT) People. Semin Oncol Nurs.

[5] Ministério da Saúde (BR) (2013). Política Nacional de Saúde Integral de Lésbicas, Gays, Bissexuais, Travestis e Transexuais.

[6] James S (2017). Report on The Experiences of Black Respondents: 2015 U.S. Transgender Survey.

[7] Page MJ, McKenzie JE, Bossuyt PM, Boutron I, Hoffmann TC, Mulrow CD (2021). The PRISMA 2020 statement: an updated guideline for reporting systematic reviews. BMJ.

[8] Peters MD (2015). The Joanna Briggs Institute reviewers’ manual 2015: methodology for JBI scoping reviews [Internet].

[9] Munn Z, Aromataris E, Tufanaru C, Stern C, Porritt K, Farrow J (2019). The development of software to support multiple systematic review types: the Joanna Briggs Institute System for the Unified Management, Assessment and Review of Information (JBI SUMARI). Int J Evid Based Healthc.

[10] Peters MDJ, Godfrey C, McInerney P, Baldini Soares C, Khalil H, Parker D, Aromataris E, Munn Z (2017). Joanna Briggs Institute Reviewer’s Manual [Internet].

[11] Franco-Rocha OY, Wheldon CW, Trainum K, Kesler SR, Henneghan AM (2023). Clinical, psychosocial, and sociodemographic factors of sexual and gender minority groups with cancer: a systematic review. Eur J Oncol Nurs.

[12] Sonnenblick EB, Lebron-Zapata L, Yang R, Dodelzon K, Sevilimedu V, Milch H (2022). Breast imaging for transgender individuals: assessment of current practice and needs. J Am Coll Radiol [Internet].

[13] Shi R, Yeoh C, Lee J, Tan KS, Yang G, Haviland K (2023). Perioperative oncology health care providers and transgender health: a singleinstitution survey to gauge attitudes, knowledge, behaviors, and education. Transgend Health.

[14] Banerjee SC, Walters CB, Staley JM, Alexander K, Parker PA (2018). Knowledge, Beliefs, and Communication Behavior of Oncology Health-care Providers (HCPs) regarding Lesbian, Gay, Bisexual, and Transgender (LGBT) Patient Health care. J Health Commun.

[15] Shires DA, Prieto L, Woodford MR, Jaffee KD, Stroumsa D (2019). Gynecologic Health Care Providers’ Willingness to Provide Routine Care and Papanicolaou Tests for Transmasculine Individuals. J Womens Health.

[16] Sutter ME, Bowman-Curci ML, Duarte Arevalo LF, Sutton SK, Quinn GP, Schabath MB (2020). A survey of oncology advanced practice providers’knowledge and attitudes towards sexual and gender minorities with cancer. J Clin Nurs.

[17] Berner AM, Hughes DJ, Tharmalingam H, Baker T, Heyworth B, Banerjee S (2020). An evaluation of self-perceived knowledge, attitudes and behaviours of UK oncologists about LGBTQ+ patients with cancer. ESMO Open.

[18] Shetty G, Sanchez JA, Lancaster JM, Wilson LE, Quinn GP, Schabath MB (2016). Oncology healthcare providers’ knowledge, attitudes, and practice behaviors regarding LGBT health. Patient Educ Couns.

[19] Ussher JM, Perz J, Allison K, Power R, Hawkey A, Dowsett GW (2022). Attitudes, knowledge and practice behaviours of oncology health care professionals towards lesbian, gay, bisexual, transgender, queer and intersex (LGBTQI) patients and their carers: a mixed-methods study. Patient Educ Couns.

[20] Unger CA (2015). Care of the transgender patient: a survey of gynecologists’ current knowledge and practice. J Womens Health.

[21] Habib L, Shirima S, Wang Y, Bidell M, Quinn G, Radix A (2023). Cognitive testing of healthcare professional and patient scales to assess affirming care for lesbian, gay, bisexual, transgender, queer, and intersex persons: the queering individual and relational knowledge scales (QUIRKS) [Internet].

[22] Zayhowski K, Park J, Boehmer U, Gabriel C, Berro T, Campion M (2019). Cancer genetic counselors’ experiences with transgender patients: a qualitative study. J Genet Couns.

[23] Gatos KC (2018). A Literature Review of Cervical Cancer Screening in Transgender Men. Nurs Womens Health.

[24] Cloyes KG, Candrian C (2021). Palliative and end-of-life care for sexual and gender minority cancer survivors: a review of current research and recommendations. Curr Oncol Rep.

[25] Kamen C (2018). Lesbian, Gay, Bisexual, and Transgender (LGBT) Survivorship. Semin Oncol Nurs.

[26] Kerr L, Fisher CM, Jones T (2021). Key Informants Discuss Cancer Care Research for Trans and Gender Diverse People. J Cancer Educ.

[27] Power R, Ussher JM, Perz J, Allison K, Hawkey AJ (2022). “Surviving Discrimination by Pulling Together”: LGBTQI Cancer Patient and Carer Experiences of Minority Stress and Social Support. Front Oncol.

[28] Pratt-Chapman ML, Murphy J, Hines D, Brazinskaite R, Warren AR, Radix A (2021). “When the pain is so acute or if I think that I’m going to die”: health care seeking behaviors and experiences of transgender and gender diverse people in an urban area. PLoS One.

[29] Sledge P (2019). From decision to incision: Ideologies of gender in surgical cancer care. Soc Sci Med.

[30] Stenzel AE, Moysich KB, Ferrando CA, Starbuck KD (2020). Clinical needs for transgender men in the gynecologic oncology setting. Gynecol Oncol.

[31] Rolle L, Zayhowski K, Koeller D, Chiluiza D, Carmichael N (2022). Transgender patients’ perspectives on their cancer genetic counseling experiences. J Genet Couns.

[32] Webster R, Drury-Smith H (2021). How can we meet the support needs of LGBT cancer patients in oncology? a systematic review. Radiography.

[33] Kerr L, Fisher CM, Jones T (2021). “I’m Not From Another Planet”: The Alienating Cancer Care Experiences of Trans and Gender-Diverse People. Cancer Nurs.

[34] Floyd MJ, Martin O, Eckloff KJ (2020). A qualitative study of transgender individuals’ experiences of healthcare including radiology. Radiography.

[35] Wilson LE, Sehovic I, Sanchez JA, Sutton SK, Kanetsky PA, Simmons VN (2016). Abstract A20: LGBTQ self-disclosure in healthcare: the need for providers to discuss LGBTQ-specific cancer education. Cancer Epidemiol Biomarkers Prev.

[36] Bristowe K, Hodson M, Wee B, Almack K, Johnson K, Daveson BA (2018). Recommendations to reduce inequalities for LGBT people facing advanced illness: ACCESSCare national qualitative interview study. Palliat Med.

[37] Squires LR, Bilash T, Kamen CS, Garland SN (2022). Psychosocial needs and experiences of transgender and gender diverse people with cancer: a scoping review and recommendations for improved research and care. LGBT Health.

[38] Gannon T, Phillips B, Saunders D, Berner AM (2022). Knowing to ask and feeling safe to tell – understanding the influences of HCP-Patient Interactions in Cancer Care for LGBTQ+ Children and Young People. Front Oncol.

[39] Hunt R, Bates C, Walker S, Grierson J, Redsell S, Meads C (2019). A systematic review of uk educational and training materials aimed at health and social care staff about providing appropriate services for LGBT+ People. Int J Environ Res Public Health.

[40] Pratt-Chapman ML, Astorino J, Goyal S, Schmit B, Yap ML, Bajaj S (2023). Radiology and radiation oncology considerations for transgender and intersex patients: a qualitative study. J Med Imaging Radiat Oncol.

[41] Burgart JM, Walters RW, Shanahan M (2022). Transgender education experiences among obstetrics and gynecology residents: a national survey. Transgend Health.

[42] Morrison SD, Chong HJ, Dy GW, Grant DW, Wilson SC, Brower JP (2016). Educational exposure to transgender patient care in plastic surgery training. Plast Reconstr Surg.

[43] Moll J, Krieger P, Moreno-Walton L, Lee B, Slaven E, James T (2014). The prevalence of lesbian, gay, bisexual, and transgender health education and training in emergency medicine residency programs: what do we know?. Acad Emerg Med.

[44] Fernandes MCL, Silva W, Tolentino TS, Araújo MJA, Joventino MLS, Silva PE (2019). Conhecimento de profissionais de enfermagem acerca da assistência à saúde dos transexuais. Rev Ciênc Saúde Nova Esperança [Internet].

[45] Callahan EJ, Sitkin N, Ton H, Eidson-Ton WS, Weckstein J, Latimore D (2015). Introducing sexual orientation and gender identity into the electronic health record: one academic health center’s experience. Acad Med.

[46] Cloyes KG, Hull W, Davis A (2018). Palliative and End-of-Life Care for Lesbian, Gay, Bisexual, and Transgender (LGBT) Cancer Patients and Their Caregivers. Semin Oncol Nurs.

[47] Chidiac C, Grayson K, Almack K (2021). Development and evaluation of an LGBT+ education programme for palliative care interdisciplinary teams. Palliat Care Soc Pract.

[48] Pratt-Chapman ML, Phillips S (2020). Health professional student preparedness to care for sexual and gender minorities: efficacy of an elective interprofessional educational intervention. J Interprof Care.

[49] Pratt-Chapman ML (2021). Efficacy of LGBTQI cultural competency training for oncology social workers. J Psychosoc Oncol.

[50] Pratt-Chapman ML (2022). Learning Outcomes of Diverse Oncology Professionals After the TEAM Cultural Competency Training. J Cancer Educ.

[51] Block RG, Sampson A, Gagliardi J, Augusto B, Santiago-Datil W, Schabath MB (2022). The LOvE ECHO Training: Developing a Web-Based LGBTQ Cultural Competency Training Module for Oncology Allied Health Professionals. J Adolesc Young Adult Oncol.

