# Supplementary material for: Assessment of knowledge in oncology about care for transgender people: a scoping review
Source: Rev Bras Enferm. 2024 Nov 29;77(Suppl 3):e20230532. doi: 10.1590/0034-7167-2023-0532 (PMC11654234; doi:10.1590/0034-7167-2023-0532)
Supplement: Translation

# Translation: Avaliação do conhecimento em oncologia acerca do atendimento às pessoas transgênero: revisão de escopo

**Authors:** Fernanda Fachetti Xavier de Almeida, Cremilson de Paula Silva, Ricardo Souza Evangelista Sant’Ana, Ruan Nilton Rodrigues Melo

**Affiliations:** I Fundação Antônio Prudente A.C.Camargo Cancer Center. São Paulo, São Paulo, Brasil.; II Universidade Federal de Alfenas. Alfenas, Minas Gerais, Brasil.; III Universidade de São Paulo. Ribeirão Preto, São Paulo, Brasil.; IV Universidade de São Paulo. São Paulo, Brasil.

**Keywords:** Pessoas Transgênero, Serviços de Saúde para Pessoas Transgênero, Neoplasias, Oncologia, Capacitação Profissional

## Abstract

**Objetivo:**

identificar as evidências disponíveis na literatura sobre os instrumentos e as metodologias utilizadas para avaliar o conhecimento de profissionais de saúde acerca do atendimento oncológico à população transgênero.

**Métodos:**

revisão de escopo, conduzida em sete bases de dados, incluindo estudos que respondessem à pergunta: qual o nível de conhecimento dos profissionais de saúde acerca do atendimento oncológico à população transgênero?

**Resultados:**

foram selecionados 41 artigos que tratavam especificamente do conhecimento dos profissionais de saúde em relação ao atendimento à população LGBTQIAPN+, especialmente à população trans. Dezoito estudos avaliaram a percepção dos pacientes a respeito do conhecimento dos profissionais, enquanto que outros estudos utilizaram instrumentos próprios para avaliação, considerando o contexto global da saúde LGBTQIAPN+.

**Conclusões:**

não há um instrumento testado e validado que avalie o conhecimento sobre saúde oncológica da população transgênero, evidenciando a necessidade da construção e validação de um instrumento focado nas necessidades dessa população.

## INTRODUÇÃO

O termo LGBTQIAPN+ e suas diversas variações, que buscam abranger a diversidade de identidades existentes, englobam um grupo heterogêneo com indivíduos de diferentes idades, experiências, origens étnicas, culturais e socioeconômicas. Essas identidades vivenciam um cotidiano de discriminações e violências simbólicas devido aos estigmas sociais. Essas dificuldades resultam em menor busca por cuidados de rotina e aumento da incidência de doenças crônicas, incluindo câncer, em comparação com a população geral^(1,2)^.

Ao abordar a comunidade LGBTQIAPN+, é essencial considerar o conceito de interseccionalidade, que abrange a sobreposição de identidades sociais e sistemas de opressão. É fundamental direcionar atenção aos grupos de maior vulnerabilidade dentro dessa minoria. Entre esses grupos, os indivíduos transgêneros emergem como particularmente marginalizados^(3)^.

O termo “transgênero” refere-se a indivíduos cuja identidade ou expressão de gênero difere da atribuída ao nascimento. A identidade de gênero é uma experiência pessoal, abrangendo o senso individual do corpo e diversas expressões de gênero, nem sempre visíveis para os outros. A ausência de coleta de dados de orientação sexual e identidade de gênero em instrumentos de saúde dificulta a avaliação do impacto real do câncer na população transgênero e LGBTQIAPN+ como um todo^(4)^. No entanto, há indícios de que as pessoas trans enfrentam desproporcionalmente fatores de risco modificáveis para o câncer, como tabagismo, obesidade, exposição a vírus associados aos comportamentos sexuais, como HPV e HIV, além da falta de serviços especializados na saúde transgênero e rastreio inadequado do câncer^(2)^.

A falta de rastreamento adequado do câncer, somada ao aumento de fatores de risco, resulta em uma maior incidência de casos diagnosticados em estágios avançados, prejudicando o prognóstico de saúde dessa população marginalizada^(4)^. A literatura destaca três questões essenciais relacionadas ao conhecimento atual sobre o câncer em pessoas LGBTQIAPN+: falta de coleta de dados sobre orientação sexual e identidade de gênero; ausência de uma força de trabalho e sistema de saúde culturalmente competentes; e escassez de informações educativas direcionadas para essa população^(4)^. A competência cultural abrange conhecimento, atitudes inclusivas e habilidades de comunicação, sendo fundamental nesse contexto^(4)^.

A Política Nacional de Saúde Integral LGBT, estabelecida em 2011 pela Portaria nº 2.836, busca combater a LGBTfobia institucionalizada, garantindo o cumprimento dos princípios de universalidade, equidade e integralidade do Sistema Único de Saúde (SUS). Contudo, suas diretrizes específicas para prevenção de cânceres em pessoas trans e travestis carecem de orientações claras sobre como deve ser realizado esse rastreamento^(5)^.

Os desafios na prevenção para a população trans incluem ainda a heteronormatividade predominante em campanhas de rastreamento de câncer, evidenciada pelo uso simbólico da fita cor-de-rosa para câncer de mama e da fita azul para câncer de próstata. Além disso, os espaços de tratamento do câncer refletem padrões de“gênero”, inadvertidamente discriminando indivíduos transgêneros^(3)^. O uso do nome social, garantido pela Política Nacional de Saúde Integral LGBT, muitas vezes encontra resistência na prática, gerando constrangimento e estresse emocional^(5)^.

A pesquisa do *National Center for Transgender Equality* revela que quase um terço dos entrevistados enfrentou assédio verbal, moral e/ou físico, ao mostrar documentos de identidade com nome ou sexo discordantes de sua apresentação de gênero, destacando a necessidade urgente de ambientes de saúde mais inclusivos e respeitosos^(6)^.

O principal desafio para a população LGBTQIAPN+ no acesso aos cuidados de saúde é o receio de discriminação por parte dos profissionais, frequentemente fundamentado em experiências negativas anteriores. A pesquisa transgênero dos EUA em 2015 revelou que 33% dos entrevistados transgêneros enfrentaram assédio verbal, recusa de tratamento ou tiveram que instruir os prestadores de cuidados sobre questões transgêneros, enquanto que 23% evitaram consultas médicas devido ao medo de maus-tratos^(6)^. A literatura destaca a falta geral de competência cultural dos profissionais de saúde em lidar com minorias sexuais e, principalmente, de gênero, sendo que a carga horária mínima e o foco restrito em temas como saúde sexual, infecções sexualmente transmissíveis e HIV nos cursos de saúde contribuem diretamente para essa deficiência^(2,4)^.

## OBJETIVO

Identificar e mapear as evidências científicas disponíveis na literatura sobre os instrumentos e as metodologias utilizadas para avaliar o conhecimento de profissionais de saúde acerca do atendimento oncológico à população transgênero.

## MÉTODOS

### Aspectos éticos

Por se tratar de estudo de revisão, não houve a necessidade de apreciação pelo Comitê de Ética em Pesquisa, de acordo com o Parágrafo Único do Art. 1º da Resolução do Conselho Nacional de Saúde nº 510/16, item VI. Ainda, ressalta-se que os estudos selecionados para a amostra final foram devidamente referenciados.

### Desenho do estudo

O estudo foi conduzido conforme a estrutura metodológica adotada pelo JBI, composta por nove etapas para *scoping review* ^(7,8)^, bem como o *checklist Preferred Reporting Items for Systematic Reviews and Meta-Analyses extension for Scoping Reviews* (PRISMA-ScR) ^(9,10)^. Ressalta-se que todos os autores realizaram treinamento no JBI sobre *scoping review* antes do início do estudo, que ocorreu no período de 06 de maio de 2023 a 22 de julho de 2023.

### Protocolo e registro

O protocolo do estudo foi registrado por meio da plataforma *Open Science Framework* (OSF), com o registro de DOI: 10.17605/ OSF.IO/EFQAN.

### Questão de pesquisa

Para a elaboração da questão de pesquisa, utilizou-se a estratégia PCC (*Population, Concept* e *Context*). Definiu-se: P – População: profissionais de saúde; C – Conceito: nível de conhecimento e C – Contexto: atendimento oncológico às pessoas transgênero. Por conseguinte, a questão norteadora da pesquisa foi: qual o nível do conhecimento dos profissionais de saúde acerca do atendimento oncológico à população transgênero?

### Estratégia de busca e fontes de dados

A estratégia de busca foi desenvolvida com a ajuda de uma bibliotecária (JDLA) da instituição da investigadora principal, utilizando descritores controlados e não controlados do *Medical Subject Headings* (MeSH), *Cumulative Index to Nursing and Allied Health Literature* (CINAHL) e Descritores em Ciências da Saúde (DeCS). Foram selecionados os descritores mais sensíveis e confiáveis. Para elaborar a estratégia final de busca, foram usados termos relacionados ao acrônimo PCC, combinados com os operadores booleanos *AND* e *OR*. Após testar as estratégias de busca nas sete bases de dados CINAHL, PubMed (*National Library of Medicine*), EMBASE (*Elsevier Science*), LILACS (Literatura Latino-Americana e do Caribe em Ciências da Saúde), Scopus (*The Search Portal for Life Sciences*), WoS (*Web of Science*) e COCHRANE (*Cochrane Central Register of Controlled Trials*), a mesma estratégia de busca foi padronizada para todas as bases: (“*Cancer Care*” *OR* “*Cancer Care Facilities*” *OR* “*Cancer*” *OR* “*Neoplasms*” *OR* “*Oncology*”) *AND* (“*Health Services for Transgender Persons*”*OR*“*Transgender Persons*” *OR* “Transgender”) *AND* (“*Professional*”).

### Critério de seleção

A busca foi realizada em 06 de maio de 2023 por dois autores independentes (FFXA, CPS). Não foram selecionados filtros de idiomas ou período de publicação. Incluíram-se todos os tipos de estudo, desde que: I) envolvessem abordagem da população transgênero; II) realizassem avaliação do nível de conhecimento/preparo dos profissionais de saúde no contexto oncológico.

### Seleção de fontes de evidência

O acesso às fontes de dados se deu por meio do Portal de Periódico da Coordenação de Aperfeiçoamento de Pessoal de Nível Superior (CAPES).

### Mapeamento e extração de dados

Os títulos e resumos recuperados nas buscas foram agrupados no banco de dados de gerenciamento de referência *EndNote Web* ^®^ para a identificação e exclusão de duplicatas. Para seleção e avaliação dos estudos da amostra, foi utilizada a plataforma *Rayyan QCRI*. Dois revisores (FFXA, CPS) realizaram a leitura e análise dos títulos e resumos, de modo independente, assegurando a realização do processo às cegas por meio do recurso *blind on* disponível no *software*, a fim de identificar os estudos pertinentes à questão norteadora desta pesquisa. As divergências quanto ao processo de avaliação dos estudos entre os revisores foram resolvidas por um terceiro revisor (RNRM). Após a busca, foram identificados os artigos que não estavam disponíveis na íntegra e, para a recuperação desses, foram realizadas até três tentativas de contato com os autores. A Figura 1 aponta a quantidade de artigos encontrados e incluídos apresentados pelo fluxograma PRISMA-ScR.

**Figura 1:** Fluxograma de busca dos estudos baseado no *Preferred Reporting Items for Systematic reviews and Meta-Analyses extension for Scoping Reviews*, 2023

### Análise e apresentação de dados

Após a análise dos estudos que compuseram a amostra final (FFXA, CPS, RNRM e RSES), foi possível categorizá-los em cinco categorias, sendo elas: Instrumentos utilizados para avaliação do conhecimento dos profissionais de saúde acerca do atendimento aos pacientes LGBTQIAPN+ com câncer (nessa categoria, foram incluídos os estudos que utilizaram algum instrumento que avaliasse o conhecimento oncológico dos profissionais de saúde acerca da população LGBTQIAPN+); Percepção de pacientes oncológicos LGBTQIAPN+ acerca do conhecimento dos profissionais de saúde (essa categoria incluiu os estudos que avaliavam qual a percepção que pacientes LGBTQIAPN+ com câncer possuíam em relação ao conhecimento dos profissionais); Formação acadêmica incipiente: dificuldades para uma assistência integral à população LGBTQIAPN+ (nessa categoria, foram incluídos os estudos que, apesar de avaliar o conhecimento, abordaram as fragilidades encontradas no sistema educacional na área da saúde); Implementação do Processo de Enfermagem: fragilidades na assistência (aqui foram incluídos estudos que abordassem os desafios da enfermagem na assistência de pessoas pertencentes a minorias sexuais e de gênero); Cuidado integral: estratégias para oferecer uma abordagem integral às necessidades e demandas dos pacientes LGBTQIAPN+ com câncer (os estudos incluídos nessa categoria abordaram informações sobre possíveis estratégias para melhora da assistência aos pacientes LGBTQIAPN+).

## RESULTADOS

As estratégias de busca permitiram encontrar 364 artigos, dos quais 81 replicados foram eliminados através do *software* gerenciador de referências *EndNote Web* ^®^. Posteriormente, os estudos restantes dessa análise inicial foram transferidos para o banco de dados elaborado no aplicativo *Rayyan QCRI*, onde foi possível eliminar outros 72 estudos duplicados. Após a avaliação do terceiro revisor, foram selecionados 74 artigos para serem avaliados na íntegra e analisados de acordo com os critérios de elegibilidade pré-estabelecidos. Desses, 41 artigos tratavam especificamente do conhecimento dos profissionais de saúde em relação ao atendimento à população LGBTQIAPN+, em especial à população trans.

A saúde psicossocial é influenciada por uma interseção complexa de fatores demográficos, clínicos e sociais nos quais os indivíduos estão incluídos^(11)^. Em comparação aos grupos heterossexuais cisgêneros com câncer, as minorias sexuais e de gênero com câncer apresentam diferenças significativas em termos sociodemográficos, psicossociais e clínicos, refletindo experiências únicas moldadas por fatores sistêmicos que marginalizam identidades *Queer* e favorecem identidades cisgênero e heterossexuais^(11)^. Isso faz com que essas minorias vivenciem o processo de saúde-doença de formas distintas, inclusive dentro dos subgrupos LGBTQIAPN+, necessitando de cuidados diferenciados^(11)^. Os estudos levantados nesta revisão abordam o conhecimento dos profissionais acerca dessas diferenças.

Em relação à localização de publicação das pesquisas, identificou-se que a América do Norte e a Europa foram as grandes responsáveis pelo conteúdo produzido. Os Estados Unidos lideraram em número de publicações, com 31 estudos (78%), seguidos pelo Reino Unido, com seis (15%), Austrália, com dois (5%), Suíça e Canadá, com um estudo cada, respectivamente.

Quanto à abordagem metodológica dos estudos, observou-se uma variedade de desenhos de pesquisa. Um total de 15 estudos foram conduzidos com abordagem qualitativa (36,59%), indicando uma forte presença de pesquisas exploratórias e descritivas. Identificaram-se 11 estudos de revisão: cinco foram classificados como revisões sistemáticas (12,20%); quatro foram classificados como revisões narrativas (9,76%); e dois foram classificados como *scoping reviews* (4,88%). Dez estudos utilizaram o delineamento transversal (24,39%) e cinco estudos foram classificados como quase-experimentais (12,20%).

Tratando-se do ano de publicação dos 41 estudos que compuseram a amostra desta revisão, houve predominância de publicações no ano de 2022, com nove artigos (21,95%), seguidos pelos anos de 2018 e 2021, com sete publicações cada (17,07%). Nos estudos analisados, o desenho metodológico mais utilizado foi o qualitativo, em 16 pesquisas (39,02%), seguido por estudos quantitativos do tipo transversal, em dez estudos (24,39%), assim como estudos de revisão, respectivamente.

Quanto à população-alvo, 20 estudos focaram apenas nos profissionais de saúde, dos quais nove abordaram apenas especialistas e/ou profissionais que atuavam em serviços de oncologia, e apenas um abordou estudantes de graduação na área da saúde. Dois estudos foram desenvolvidos em centros de tratamento de câncer, enquanto que os demais não fizeram distinção quanto ao nível de atenção à saúde em que os profissionais atuavam. Foram incluídos 13 estudos envolvendo a população LGBTQIAPN+: seis deles abordaram apenas pacientes oncológicos, sendo que um focou exclusivamente nos sobreviventes de câncer; dois estudos também incluíram os cuidadores desses pacientes; um estudo envolveu defensores e pesquisadores de questões relacionadas à população LGBTQIAPN+. Apenas seis estudos focaram exclusivamente na população transgênero, sendo quatro deles no contexto oncológico, o que evidencia a escassez de pesquisas que abordem as especificidades dessa população. Dois estudos abordaram tanto a perspectiva dos profissionais quanto a dos pacientes. Embora alguns estudos tenham mencionado desafios relacionados às dificuldades financeiras e à cobertura dos planos de saúde, nenhum especificou se as discussões tratavam de serviços de saúde públicos ou privados.

**Quadro 1:** Distribuição dos estudos segundo o código do artigo, o autor principal, o ano de publicação, a categoria na qual foram incluídos e as principais recomendações encontradas, 2023

Código/autor/ano	Categoria do estudo	Principais recomendações
A1/ Banerjee *et al*., 2018	Categoria 1	Recomenda-se um inquérito de avaliação de necessidades para identificar as dificuldades dos profissionais na comunicação com pacientes LGBT e suas necessidades, visando desenvolver um treinamento de sensibilidade.
A2/ Habib *et al*., 2023	Categoria 1	Novas pesquisas para aprimorar escalas que permitam comparar as experiências de provedores e pacientes com cuidados de saúde afirmativos para a população LGBTQI. As escalas deste estudo precisam ser avaliadas para possíveis melhorias, tornando-as menos cansativas e permitindo o relato de variações nas experiências de cuidado pelos profissionais.
A3/ Shi *et al*., 2023	Categoria 1	É necessário que as instituições avaliem a competência cultural do corpo clínico perioperatório em relação à saúde transgênero, especialmente dentro de determinados grupos demográficos.
A4/ Shires *et al*., 2019	Categoria 1	Incluir a formação em cuidados de saúde inclusivos para pessoas transgêneros como parte rotineira da formação de todos os profissionais de saúde, abordando também as atitudes pessoais.
A5/Sonnenblick *et al*., 2022	Categoria 1	Na prática de imagens mamárias, há uma necessidade substancial de registrar informações sobre transgêneros e outras informações não conformes com o gênero.
A6/ Sutter *et al*., 2020	Categoria 1	É importante enfatizar continuamente a importância da orientação sexual e da coleta de dados de identidade de gênero para apoiar os esforços educativos e, assim, melhorar a qualidade dos cuidados prestados a indivíduos LGBTQ+ ao longo do *continuum* do câncer.
A7/ Berner *et al*., 2020	Categoria 1	Deve-se integrar a conscientização sobre as necessidades de cuidados LGBTQ+ na oncologia aos currículos de pós-graduação em oncologia médica e clínica. Recomenda-se criar um repositório *online* de material educacional existente, complementado por conteúdo específico para o Reino Unido. É crucial realizar pesquisas de alta qualidade para coletar dados sobre orientação sexual e identidade de gênero, visando entender as diferenças, os riscos e os resultados do câncer nessas populações.
A8/ Shetty *et al*., 2016	Categoria 1	São necessárias grandes pesquisas nacionais para determinar se o conhecimento, as atitudes e os comportamentos práticos identificados neste estudo representam os prestadores de cuidados de saúde nos Estados Unidos. Uma amostra nacionalmente representativa é crucial para desenvolver formação e currículos que abordem as lacunas identificadas.
A9/ Zayhowski *et al*., 2019	Categoria 1	As descobertas deste estudo apontam oportunidades para o campo do aconselhamento genético melhorar os serviços para pacientes transexuais, relatando situações distintas que podem surgir na clínica com esses pacientes e fornecendo recomendações de treinamento para conselheiros genéticos.
A10/ Ussher *et al*., 2022	Categoria 1	São necessárias mudanças sistêmicas para superar as barreiras na prestação de cuidados oncológicos culturalmente competentes para pacientes LGBTQI. Assim, recomenda-se incluir conteúdo tanto para a comunidade LGBTQI em geral quanto específico para cada subgrupo dentro dessa comunidade e nos currículos de educação e treinamento profissional.
A11/ Unger *et al*., 2015	Categoria 1	Devem ser feitos esforços para educar os formandos sobre os aspectos importantes dos cuidados transgênero e devem ser publicadas diretrizes abrangentes para os prestadores de serviço.
A12/ Gatos *et al*., 2018	Categoria 2	Investir em pesquisas que analisem as experiências e necessidades dos homens transexuais para que consiga chegar a recomendações e diretrizes específicas para essa população.
A13/ Cathcart *et al*., 2020	Categoria 2	Avaliar o nível de conforto de um paciente com discussões sobre sexo e sexualidade no início e periodicamente, a fim de garantir uma qualidade de vida sexual satisfatória ao paciente.
A14 / Kamen *et al*., 2019	Categoria 2	É necessário realizar estudos focados nas experiências individuais de pacientes transexuais, não binários e negros com diagnóstico atual de câncer, além de estudos de métodos mistos para examinar as disparidades no cuidado oncológico para pacientes LGBTQI.
A15/ Cloyes; Candrian, 2021	Categoria 2	Os serviços de cuidados paliativos devem coletar dados sensíveis sobre orientação sexual e identidade de gênero dos pacientes. Além disso, é fundamental fornecer formação e educação adequadas sobre as necessidades individuais dos pacientes LGBTQ+ e criar ambientes seguros e representativos para essas populações vulneráveis.
A16/ Kamen, 2018	Categoria 2	Assegurar a competência cultural entre os profissionais de saúde acerca da população LGBT; ter serviços de referência para acolhimento; analisar e rever os programas de apoio social; e realizar educação através de organizações comunitárias.
A17/ Kerr *et al*., 2021	Categoria 2	É necessário a criação de programas de educação em parcerias com organizações de apoio a saúde transgênero, a criação de políticas inclusivas e melhora na coleta de dados para epidemiologia do câncer em pessoas transgênero.
A18/ Power *et al*., 2022	Categoria 2	Recomenda-se investir em ambientes acolhedores e seguros, focando na avaliação e no aumento do conhecimento dos profissionais sobre saúde LGBTQI. Também sugere a importância de ter profissionais que fazem parte dessa comunidade como provedores de cuidado.
A19/Pratt-Chapman *et al*., 2021	Categoria 2	Recomenda-se que haja explicações claras e conversas abertas com os pacientes, além da formulação de diretrizes de rastreamento específicas para as minorias sexuais e de gênero.
A20/ Sledge, 2019	Categoria 2	O estudo recomenda que as instituições criem ações e pesquisas que colaborem para o desenvolvimento do cuidado centrado no paciente, a fim de permitir que eles consigam expressar suas vontades e desejos.
A21/ Stenzel *et al*., 2020	Categoria 2	Fornecer formação educacional aos médicos sobre cuidados de gênero e minorias sexuais, reconhecendo as disparidades no sofrimento em populações vulneráveis.
A22/ Rolle *et al*., 2022	Categoria 2	Recomendam-se melhorias no sistema de formação educacional dos profissionais de saúde, além de investigações a respeito do rastreio, da vigilância e das experiências de homens transexuais em relação ao câncer.
A23/ Webster *et al*., 2021	Categoria 2	Educação em relação a linguagens e pronomes adequados e as propostas de educação em oncologia e radioterapia sob a ótica das disparidades; criação de grupos de suporte; coletas de dados sobre orientação sexual e identidade de gênero e investimentos em pesquisas na área.
A24/ Kerr *et al*., 2021	Categoria 2	Os enfermeiros oncológicos precisam refletir sobre seus próprios pressupostos em relação às questões de gênero e estreitar laços para uma comunicação aberta com seus pacientes.
A25/ Floyd, *et al*., 2020	Categoria 2	É necessário a melhora dos currículos de graduação e pós-graduação com inclusão de temas de diversidade sexual e de gênero para todos os profissionais de saúde, além da adoção de linguagem inclusiva e ambiente acolhedor.
A26/ Lauren *et al*., 2016	Categoria 2	Revisão de bases curriculares dos cursos de medicina e de saúde e aumento das interações com essa população desde a comunicação até as relações com as pessoas.
A27/ Bristowe *et al*., 2018	Categoria 2	Usar a linguagem adequada, explorar os cuidados com a região íntima de pacientes transexuais, auxiliar na criação de políticas de apoio e inclusão e aumentar a visibilidade de pessoas LGBT em materiais institucionais.
A28/ Squires *et al*., 2022	Categoria 2	Os estudos devem aprofundar mais sobre as questões biopsicossociais com colaborações interinstitucionais, maior foco na sigla mais vulnerável que são das pessoas transexuais. Criar um ambiente acolhedor e promover a educação específica para os pacientes e profissionais.
A29/ Gannon *et al*., 2022	Categoria 2	Recomenda-se o uso de terminologias e linguagem apropriadas, além da divulgação e educação sobre o tema. Além disso, questões institucionais de acolhimento e pertencimento devem ser implementadas para melhora da experiência do paciente.
A30/ Burgart *et al*., 2022	Categoria 3	Implementar um método de distribuição mais uniforme e confiável, enviando pesquisas diretamente aos residentes ou incluindo um número limitado de perguntas no exame anual de serviço para todos os examinandos de residentes. Mensurar esses dados poderia melhorar a educação de residentes, especialmente após a implementação de intervenções específicas.
A31/ Hunt *et al*., 2019	Categoria 3	É necessária uma melhor formação do pessoal de saúde e de assistência social. Devem ser desenvolvidos e depois avaliados materiais de formação que incorporem mais técnicas de mudança de atitude e comportamento baseadas em evidências para garantir a sua eficácia junto do pessoal de saúde e de assistência social em uma ampla variedade de contextos.
A32/PrattChapman *et al*., 2023	Categoria 3	Treinamento de competência dos radiologistas para o atendimento da população transgênero e intersexual abordando terminologia, exame físico, adaptações da radioterapia, potenciais efeitos colaterais para pacientes com transtornos globais do desenvolvimento e intersexuais, experiências pessoais no atendimento a essas minorias, recomendações clinicas e coordenação de cuidados.
A33/ Damascos *et al*., 2018	Categoria 4	Investigação ampliada sobre questões LGBT, incluindo identidades interseccionais e determinantes sociais que afetam a prevenção, rastreamento, tratamento e sobrevivência do câncer. Além disso, é essencial aumentar o acesso aos rastreamentos de câncer e a educação inclusiva para todas as pessoas LGBT, promovendo comportamentos de saúde positivos. Formações baseadas na humildade cultural e na interseccionalidade são necessárias para capacitar os profissionais a fornecer cuidados compassivos às pessoas LGBT com câncer.
A34/ Callahan *et al*., 2015	Categoria 4	As lições aprendidas para melhorar os cuidados aos pacientes LGBT podem ser aplicadas aos esforços para melhorar os cuidados a todos os grupos marginalizados. Para fazer mudanças na prestação de cuidados de saúde, os indivíduos e as instituições precisam assumir um compromisso a longo prazo com a mudança.
A35/ Cloyes *et al*., 2018	Categoria 5	Implementação do tema diversidade nos currículos de formação; criação de recursos de apoio e orientação; garantia da avaliação de conhecimento; e treinamento contínuo de competências.
A36/ Chidiac *et al*., 2021	Categoria 5	Desenvolver treinamentos educativos e avaliação de conhecimento em parceria com diversos setores e equipes no âmbito da saúde.
A37/PrattChapman, 2022	Categoria 5	Desenvolver estudos que utilizem medidas validadas para avaliação de competência cultural e conhecimento dos profissionais acerca da oncologia aplicada à população transgênero.
A38/PrattChapman, Phillips, 2020	Categoria 5	Desenvolver estratégias de educação continuada visando diversificar e preparar os profissionais para o atendimento das demandas dos pacientes da população LGBT, além da importância da avaliação do conhecimento desses mesmos indivíduos.
A39/PrattChapman. 2021	Categoria 5	Promover oportunidades de autorreflexão entre os profissionais, além de estruturar estratégias educativas que sejam interativas para melhorar o conhecimento.
A40/ Block *et al*., 2022	Categoria 5	As estratégias de implementação de treinamento, além de serem validadas com profissionais, recomendam que sejam validadas com pacientes de diferentes orientações sexuais e identidades de gênero para garantir maior representatividade nos treinamentos.

## DISCUSSÃO

### Instrumentos utilizados para avaliação do conhecimento dos profissionais de saúde acerca do atendimento ao paciente LGBTQIAPN+ com câncer

Onze estudos selecionados tiveram como foco a avaliação do conhecimento e/ou percepção dos profissionais de saúde.

A maioria dos pesquisadores estruturou seus próprios instrumentos de avaliação para atingir objetivos específicos. Sonnenblick *et al*. (2022)^(12)^ criaram um instrumento para avaliar a prática e as necessidades na área da imagem mamária para indivíduos transgêneros, enquanto que Shi *et al*. (2023)^(13)^ focaram na atitude, conhecimento e comportamento de profissionais de saúde perioperatórios em relação à saúde de pessoas trans.

Outros estudos utilizaram questionários personalizados para examinar a importância do conhecimento dos profissionais de saúde sobre as necessidades específicas de pacientes LGBTQIAPN+, ressaltando a necessidade de uma atenção mais focada em comunicação, crenças e comportamento dos provedores de saúde oncológica para essa população^(14 ^–^ 20)^.

Habib *et al*. (2023)^(21)^ validaram um instrumento específico para avaliar a atenção afirmativa aos pacientes LGBTQIAPN+. O estudo desenvolveu duas escalas, QUIRKS-*Provider* e QUIRKS-*Patient*, para avaliar o ambiente e os comportamentos clínicos. A escala para profissionais avaliou conhecimento objetivo e preparação autorrelatada, enquanto que a escala para pacientes avaliou experiências de cuidado e satisfação.

O instrumento *Lesbian, Gay, Bisexual, and Transgender Development of Clinical Skills Scale* (LGBT-DOCSS) é a única escala validada para avaliar conhecimentos, habilidades, atitudes e preparação de autorrelatos pelos profissionais de saúde no cuidado de pessoas LGBT. Essa escala é usada por Habib *et al*. (2023)^(21)^ e outros estudos, mas é genérica e não aborda especificamente o câncer, nem as subpopulações, como pessoas transgênero.

Alguns estudos optaram por abordagens qualitativas, como entrevistas semiestruturadas, para obter uma visão mais subjetiva. Zayhowski *et al*. (2019)^(22)^ exploraram experiências de conselheiros genéticos oncológicos no atendimento a pacientes transgêneros. Vários estudos destacaram a necessidade de treinamento contínuo e educação para melhorar o conhecimento e atitudes dos profissionais de saúde^(17 ^–^ 19)^. Isso sublinha a importância de incluir esses temas nos currículos de treinamento e implementar estratégias educacionais específicas e contínuas para uma prestação de cuidados mais inclusiva e sensível às necessidades dessa população.

Alguns estudos identificaram atitudes desafiadoras e comportamentos que refletem falta de sensibilidade em relação a pacientes LGBTQIAPN+, como a relutância em fornecer cuidados rotineiros, como exame citopatológico do colo do útero para indivíduos trans masculinos^(13,16)^.

Estudo com delineamento qualitativo, realizado por meio de entrevista, destacou desafios interpessoais na interação entre profissionais de saúde e pacientes transgêneros, indicando a necessidade não apenas de conhecimento técnico, mas também de habilidades interpessoais específicas para fornecer um atendimento inclusivo^(22)^.

### Percepção de pacientes oncológicos LGBTQIAPN+ acerca do conhecimento dos profissionais de saúde

Nessa categoria, 18 estudos que abordam o conhecimento dos profissionais de saúde sob a ótica dos pacientes com câncer foram incluídos para análise. Todos os estudos incluídos nessa categoria indicaram a necessidade de formação adequada para os profissionais de saúde^(3,23,24,25,26,27,28,29,30,31,32,33,34,35,36,37,38)^. A maioria destacou a fragilidade no conhecimento específico, habilidades e abordagem à população LGBTQIAPN+, especialmente à população transgênero^(3,23,24,25,26,28,29,30,31,33,34,35,36)^. Os estudos apontaram que os profissionais de saúde estão despreparados para atender aos pacientes, oferecendo assistência de baixa qualidade devido à falta de informações completas e pertinentes, o que constitui uma barreira para a obtenção de um cuidado integral^(3,23,28,30,31,33,36,38)^. Além disso, os estudos salientaram que os profissionais não possuem conhecimento sobre como questionar os pacientes a respeito de sua identidade de gênero e sexualidade^(3,23,26,28,30,32,33,34,35,38)^. A falta de compreensão sobre o conceito de transexualidade, o processo de transição e a má interpretação de sexualidade, gênero, suposições e questões relacionadas à saúde foram identificados como barreiras de acesso para a população transgênero, resultando em experiências negativas durante a busca por cuidados de saúde, fazendo com que se sintam “invisíveis”e com suas necessidades não atendidas^(32,34,35)^.

Além disso, as pesquisas analisadas indicam que a falta de sensibilização, empatia e compreensão das necessidades individuais dos pacientes LGBTQIAPN+ contribui para o surgimento de medo, angústia, estresse emocional e psicológico^(3,23,24,25,26,28,32,33)^. Isso, por sua vez, cria barreiras para o acesso aos serviços de saúde, dificulta a obtenção de informações precisas sobre o percurso terapêutico e pode afetar negativamente a interação com os profissionais de saúde^(3,23,24,25,26,27,28,30,32,34,35,38)^. Essa interação já é comprometida pelo diagnóstico de uma doença que pode interferir na continuidade da vida e trazer consequências emocionais, físicas, sociais e espirituais^(36,38)^.

A discriminação e o preconceito historicamente vivenciados pelos participantes também foram apontados nos estudos como fatores agravantes na interação dos pacientes com os profissionais^(3,24,25,26,27,30,32,33,34,35,36)^. Esse preconceito e estigma intrínsecos na sociedade, juntamente com a falta de conhecimento, constituem obstáculos individuais e institucionais^(24)^. Para minimizar os impactos desse fato histórico, estudos afirmam a necessidade de os prestadores de cuidados de saúde deixarem explícito que todas as formas de discriminação e preconceito não serão toleradas^(25,28,32,35,38)^.

Nesse contexto, o estudo de Kamen *et al*. (2019), com abordagem qualitativa e que aborda sobre as experiências e recomendações de pacientes lésbicas, gays, bissexuais, transgêneros e *queer* (LGBTQ) com câncer, enfatiza que uma abordagem integral aos pacientes deve incluir a negociação da divulgação da orientação sexual e identidade de gênero com base na segurança dos pacientes^(3,24,25,32,36,38)^. Outro estudo de revisão conduzido por Cloyes e Candrian (2021) apontou que os efeitos das disparidades históricas e estruturais enfrentadas pelos pacientes LGBTQIAPN+ podem ser minimizados pelo conhecimento e habilidades necessárias para fornecer cuidados competentes e centrados na individualidade de cada pessoa. Isso inclui a participação de familiares e parceiros como agentes no processo de cuidado^(24)^.

O apoio dos familiares, grupos inclusivos de apoio e especialmente a presença dos companheiros durante o percurso de diagnóstico até o prognóstico foram destacados na maioria dos estudos como atenuantes dos impactos negativos relacionados ao preconceito e à falta de conhecimento por parte dos profissionais^(3,24,25,27,31,32,33,34,35,36)^. Além disso, foi observado que a exclusão dos parceiros na assistência à saúde resulta na perda de dignidade dos pacientes, interferindo negativamente em todas as esferas de suas vidas^(28,29,32)^.

Seguindo esses princípios, os estudos indicaram que os participantes se sentiam desconfortáveis em grupos de apoio tradicionais, experimentando sentimentos como solidão e tristeza, além de não se sentirem acolhidos para falar livremente sobre questões relacionadas à saúde sexual e morbidade sem medo de discriminação^(3,24,25,26,32,33)^. A falta de conhecimento em relação às necessidades individuais e específicas dos pacientes LGBTQIAPN+ com câncer resulta em alterações emocionais e psicológicas, como destacado em estudo conduzido por Webster e Drury-Smith (2021), que evidenciou a vulnerabilidade nos serviços de saúde por não oferecerem atendimento psicológico adequado aos pacientes LGBTQIAPN+^(32)^.

Além disso, por meio da análise dos estudos^(3,23,24,25,26,27,28,29,30,31,32,33,34,35,36,37,38)^, compreende-se a necessidade de ações de educação permanente e contínua para os profissionais de saúde, visando minimizar os impactos das disparidades históricas e melhorar a prestação de serviços às pessoas LGBTQIAPN+ com câncer, cujo intuito consiste em oferecer um cuidado de forma integral, digno, respeitoso e qualificado.

### Formação acadêmica incipiente: dificuldades para uma assistência integral à população LGBTQIAPN+

Três estudos analisaram o conhecimento de profissionais de saúde que atuam no cuidado ao paciente LGBTQIAPN+. Os dados demonstram que, apesar dos investimentos em formação profissional por programas de graduação e/ou pós-graduação, os pacientes ainda se sentem vulneráveis em relação à assistência oferecida pelos profissionais^(39,40)^.

Em entrevistas realizadas com 16 profissionais que trabalham em serviços oncológicos, pode-se identificar uma fragilidade no processo formativo, conhecimento e confiança relacionados aos aspectos multidimensionais do atendimento a pessoas transexuais diagnosticadas com câncer^(39)^. Durante a formação dos estudantes, foi destacado que as informações sobre cuidados à população LGBTQIAPN+ são limitadas e não fornecem subsídios concretos para a execução de um cuidado qualificado e centrado nas necessidades desse público.

Além disso, estudo realizado por Burgart *et al*. (2022) revelou que a maioria dos médicos residentes não se sentia preparada para oferecer atendimento à população transgênero, com muitos relatando pouco acesso a conteúdos relacionados durante a formação acadêmica^(41)^. Unger (2015) também identificou que a maioria dos residentes em obstetrícia e ginecologia carece de formação específica em cuidados de saúde para pessoas transgêneros, independentemente do tempo de prática^(20)^.

Esses achados corroboram outros estudos que demonstram a fragilidade no processo formativo, contribuindo para lacunas no estabelecimento de vínculos com os pacientes e dificuldades na oferta de atendimento integral^(42,43)^. Essa lacuna de conhecimento em relação ao atendimento é também atribuída à falta de treinamento e educação continuada com foco em estratégias de abordagem à população transgênero^(44)^.

Diante disso, torna-se fundamental incluir esta temática nos currículos de graduação e pós-graduação para promover experiências positivas aos pacientes, aprimorando as ações de rastreamento do câncer, a qualidade de vida e aumentando a interação e a satisfação com os serviços de saúde, bem como com a assistência oferecida pelos profissionais que atuam na área oncológica.

### Implementação do Processo de Enfermagem: fragilidades na assistência

Dois artigos abordaram a integração da orientação sexual e identidade de gênero nos cuidados de saúde, destacando aspectos cruciais na fragilidade na assistência, mas com enfoques distintos.

Damaskos *et al*. (2018) ressaltam a importância de considerar a interseccionalidade ao atender pacientes LGBTQIAPN+ com câncer e discutem as fragilidades na assistência devido à falta de reconhecimento dessas múltiplas identidades^(1)^. Reconhecem que a experiência do câncer é moldada por uma variedade de aspectos, como raça, classe social e outros fatores. Uma abordagem sensível a esses fatores é fundamental para entender de forma integral as necessidades e desafios enfrentados por esses pacientes. A negligência desses aspectos pode resultar em lacunas significativas nos cuidados de saúde.

Callahan *et al*. (2015) corroboram essa necessidade, enfatizando a importância de incluir informações sobre orientação sexual e identidade de gênero nos registros de saúde eletrônicos para garantir uma prestação de cuidados mais abrangente^(45)^. O estudo aborda a implementação prática da coleta de dados de orientação sexual e identidade de gênero, destacando os desafios e as oportunidades de incorporar informações sensíveis ao contexto LGBTQIAPN+ nos registros de saúde eletrônicos^(45)^. Ressaltam que a falta de infraestrutura adequada e a cultura institucional podem representar obstáculos para essa implementação, necessitando de adaptações.

Esses estudos enfatizam a importância de uma abordagem inclusiva e sensível às necessidades específicas dos pacientes LGBTQIAPN+ com câncer para melhorar a qualidade dos cuidados de saúde fornecidos.

### Cuidado integral: estratégias para oferecer uma abordagem integral às necessidades e demandas dos pacientes LGBTQIAPN+ com câncer

Nessa categoria, somente dois artigos focaram na capacitação dos profissionais para prestação de cuidados paliativos e de fim de vida aos pacientes LGBTQIAPN+ com câncer, enquanto que os demais abordaram treinamentos mais amplos.

Cloyes *et al*. (2018) não realizaram nenhuma intervenção prática, mas oferecem uma revisão abrangente da literatura, destacando desafios e lacunas nos cuidados paliativos para pacientes LGBTQIAPN+, reunindo recomendações para a prática cotidiana dos profissionais, além de fontes de recursos de apoio disponíveis que visam promover cuidados paliativos e de fim de vida mais eficazes e inclusivos^(46)^. Já Chidiac *et al*. (2021) desenvolveram e implementaram um programa educacional para equipes interdisciplinares de cuidados paliativos que consistia em *workshop* de uma hora e meia, visando melhorar a competência cultural LGBTQIAPN+^(47)^. A intervenção foi realizada em quatro instituições do Reino Unido, com um total de 145 indivíduos (profissionais de saúde e assistentes sociais), e foi avaliada através do processo de oito etapas de Kotter para liderar mudanças. Um desenho pré-pós-teste de grupos quase-experimentais não equivalentes foi usado para mensurar o impacto da intervenção e mostrou um aumento significativo nos níveis relatados de conhecimento, confiança e conforto com questões, necessidades e terminologia relacionadas à população LGBTQIAPN+ e cuidados paliativos após a participação no treinamento.

Assim como Cloyes *et al*. (2018), Radix e Maingi (2018) também reuniram fontes confiáveis de recursos de apoio disponíveis para promover um cuidado de qualidade para as minorias sexuais e de gênero^(2)^. Os pesquisadores revisaram ainda as intervenções propostas e desenvolvidas, até o momento do estudo, para melhorar o conhecimento sobre a saúde LGBT. Os treinamentos levantados variaram de 45 minutos a dez horas; muitos utilizaram formato de palestra, no entanto outros incluíram estudos de caso, vídeos e/ou painéis LGBT.

Apenas um artigo teve como alvo principal a introdução de um componente de competência cultural LGBTQIAPN+ em um currículo interprofissional para estudantes de saúde. Pratt-Chapman e Phillips (2020) promoveram um simpósio de oito horas na Universidade George Washington, nos EUA, buscando preparar melhor estudantes e professores profissionais de saúde para cuidar de pacientes de minorias sexuais e de gênero^(48)^. Os pesquisadores utilizaram a escala LGBT-DOCSS para comparar o conhecimento, as atitudes e a preparação clínica dos alunos pesquisados, bem como o valor percebido da aprendizagem interprofissional antes e depois do simpósio. Além disso, também compararam os resultados pós-teste dos alunos participantes com os de um grupo interprofissional que não compareceram ao simpósio. Em contraste com o grupo de comparação, os participantes do simpósio no pós-teste obtiveram pontuações mais altas nos objetivos de aprendizagem, nas atitudes e no conhecimento dos fatores LGBT-DOCSS e no valor percebido da aprendizagem interprofissional, conforme mensurado por quatro itens da Escala de Aprendizagem Interprofissional. Esse resultado reforça o benefício de uma maior integração curricular de conteúdos de saúde de minorias sexuais e de gênero através da aprendizagem interprofissional para garantir a preparação de todos os profissionais.

Pratt-Chapman também realizou a implementação de um programa de treinamento de competência cultural para assistentes sociais oncológicos^(49)^, que consistiu em uma oficina com conteúdo didático e interativo ministrada para uma amostra de 26 assistentes sociais, além da implementação do treinamento cultural *Together for Education About Cancer in Minorities* (TEAM)^(50)^. O treinamento TEAM foi desenvolvido para melhorar a equidade na saúde em organizações de tratamento do câncer, orientando equipes de alunos interprofissionais através do planejamento e implementação de melhorias de qualidade, para promover cuidados oncológicos equitativos, acessíveis e centrados no paciente. Os componentes do treinamento incluíram: um curso *online* individualizado de cinco horas; três sessões virtuais de assistência técnica para ajudar as equipes a se preparar para a parte presencial do treinamento; um *workshop* presencial de dois dias e meio com foco no desenvolvimento de um plano de ação organizacional para melhorar um serviço relacionado ao câncer para ser mais culturalmente sensível e equitativo; e três sessões virtuais de assistência técnica para ajudar a enfrentar os desafios de implementação do plano de ação. O estudo comparou mudanças na competência cultural autorrelatada medida pela Avaliação de Competência Cultural (CCA), LGBT-DOCSS e Escala de Socialização e Valorização Interprofissional (ISVS). O objetivo principal do estudo foi avaliar as mudanças na competência cultural autorrelatada, e o objetivo secundário foi examinar as mudanças na avaliação interprofissional desde o início até a pós-intervenção.

Block *et al*. (2022) abordam a competência cultural no contexto específico da fertilidade e focam na população de jovens adultos com câncer^(51)^. A equipe *The Enriching Communication skills for Health professionals in Oncofertility* (ECHO) criou um módulo educacional em resposta às necessidades dos profissionais de saúde aliados à oncologia para fornecer cuidados inclusivos e afirmativos a pacientes jovens adultos identificados como LGBTQ com câncer.

Os artigos reunidos nesta seção destacam uma convergência notável no reconhecimento da importância de estratégias educacionais e de treinamento para aprimorar a competência cultural e a sensibilidade à diversidade entre profissionais de saúde que atendem pacientes LGBTQIAPN+ com câncer. As intervenções variaram desde programas educacionais específicos, como o treinamento cultural TEAM^(50)^, até módulos de treinamento *online*, como o *LOvE ECHO Training* ^(51)^. A avaliação quantitativa predominante por meio de questionários antes e depois das intervenções permitiu a mensuração das mudanças nas atitudes e conhecimentos dos participantes.

Os estudos produzidos e a tendência de produção científica na área revelam que as principais lacunas existentes em relação à saúde oncológica da população transgênero começam na base educacional. Identidade de gênero e orientação sexual são assuntos pouco ou nada abordados em cursos básicos de formação e, em menor número, nos cursos superiores da área da saúde. Isso faz com que os profissionais entrem no mercado de trabalho ainda com muitos preconceitos e inseguranças em relação à saúde de populações vulneráveis. Quando se trata da oncologia, os dados produzidos ainda são voltados para o binarismo de identidades de gênero cisgêneros, e há uma carência de teorias, modelos e programas aprofundados que explorem as experiências individuais sobre a doença nessa população, o que acaba gerando ainda mais desafios para o avanço da saúde transgênero no âmbito oncológico.

Além disso, a falta de conhecimento epidemiológico evidenciado em todos os estudos sobre como o câncer se comporta em populações vulneráveis específicas dificulta a obtenção de necessidades de saúde individuais e centradas no paciente.

É importante que as pesquisas científicas incluam cada vez mais em seus estudos coletas de dados acerca da identidade de gênero e orientação sexual de pessoas com câncer, para, assim, conseguir dimensionar os impactos causados devido às repercussões do câncer e seus tratamentos em populações vulneráveis, como a população transgênero.

### Limitações do estudo

Pode-se considerar como uma limitação desta pesquisa o fato de não ter conseguido acesso a 13 estudos, pois não estavam disponíveis na íntegra, mesmo após as três tentativas de contato com os autores.

### Contribuições para a área da saúde, enfermagem ou políticas públicas

Os estudos, assim como instrumentos direcionados a pessoas transgênero, residem em reconhecer que os cuidados oferecidos no contexto da enfermagem oncológica podem diferir significativamente dos outros membros da comunidade LGBTQIAPN+. O levantamento dessas necessidades e desafios é fundamental para promover a inclusão e a equidade no cuidado oncológico, podendo proporcionar um ambiente mais acolhedor e seguro para as pessoas transgênero, contribuindo para a redução das desigualdades. Isso busca melhorar os atendimentos à população transgênero e, consequentemente, seu acesso aos serviços de saúde. Nesse sentido, é possível vislumbrar as contribuições da presente pesquisa no que tange ao compromisso assumido pela enfermagem no contexto global para o alcance dos Objetivos de Desenvolvimento Sustentável (ODS) determinados pela Organização das Nações Unidas (ONU), com destaque para o ODS 3, que visa assegurar uma vida saudável e promover o bem-estar para todas as pessoas, em todas as idades.

## CONCLUSÕES

Apenas seis estudos foram direcionados exclusivamente a pessoas transgênero, o que evidencia a necessidade de ampliar a pesquisa e o conhecimento sobre as questões relacionadas ao câncer entre essa população. Além disso, não foi possível evidenciar instrumentos testados e validados para mensurar o conhecimento da saúde oncológica da população transgênero, o que demonstra uma lacuna significativa diante da literatura consultada, destacando a urgência de desenvolver e validar instrumentos específicos que possam fornecer uma avaliação precisa do conhecimento dos profissionais de saúde sobre as necessidades específicas da população transgênero no contexto do câncer.

É importante destacar a necessidade de outras pesquisas direcionadas à população transgênero no âmbito da oncologia, expandindo o conhecimento e ampliando a conscientização sobre os desafios únicos dessa comunidade, contribuindo para a melhoria dos sistemas de saúde e instrumentalizando os profissionais para fornecer cuidados equânimes e culturalmente sensíveis.

Portanto, a construção e a validação de um instrumento específico para avaliar o conhecimento da saúde oncológica da população transgênero são etapas de grande relevância para garantir a prestação de cuidados de saúde de forma inclusiva e sensível a essa comunidade culturalmente vulnerabilizada. Esse instrumento pode facilitar o desenvolvimento de intervenções e programas de treinamento direcionados e eficazes, contribuindo para a redução das disparidades no acesso e na qualidade dos cuidados oncológicos para pessoas transgênero.
